# Excerpta

**Published:** 1880-03

**Authors:** 


### EXCERPTA.

Doctor vs. Apothecary.—It is a notorious fact that many druggists use the prescriptions sent in by physicians over and over again. This they do to their own advantage and the detriment of the physicians. It is very well known, also, that druggists themselves prescribe not at all rarely, especially for venereal disease. It is a fact, in the third place, that druggists do not always put in the drugs exactly as given, or else they substitute poor drugs, and that they charge so enormously for the prescription as to make it difficult for the patient to pay both druggist and doctor.

These are old abuses, but they have recently been commented on again, and very strongly, by the New Orleans *Medical and Surgical Journal*, and the Louisville *Medical News*.

Now, the physician is the best friend the druggist has, and it will be wise for the latter to bear the fact in mind. There is a limit to the imposition to which the profession will submit. It is suggested by the *News* that a co-operative society of physicians, buying its own medicines in convenient and elegant form at wholesale prices, and furnishing them at cost to its members, might be a means of saving both physicians and patients no little money. Whether such a scheme be practicable or not, it is certain that something will eventually be done in this direction unless the druggist mends his ways.—*N. Y. Medical Record.*

A Crusade Against Quackery.—The Illinois State Board of Health held a meeting, February 5, at Chicago, mainly to consider the charges of unprofessional and dishonorable conduct brought against parties who have the certificates of the board. The board has not heretofore been able, for want of time, to take up many of these cases, but now intends to fully carry out the letter and spirit of the law, which is designed to protect the public against all sorts of professional ignorance, quackery and crime, and this is the inauguration of aggressive work in this direction. The moral force of the statute to regulate the practice of medicine has, it is stated, operated to the satisfaction of all interested, and it has been the main dependence of the board and the friends of the law heretofore. The board has fully prepared itself for an active campaign.

The Doctors in Memphis during the Epidemic of Yellow Fever.—*N. Y. Medical Record:* In 1878 all the homeopaths —four in number—ran away when the plague came. Of the forty-six regulars, ten followed in their wake. Of the thirty-six who remained, twenty-eight were attacked with the fever and fourteen died. Eight already had had the disease and were not attacked, although on duty day and night. This fact corroborates the belief that one attack gives immunity from a second.—*Louisville Medical News.*

Jaborandi in Dropsy.—The Boston *Medical and Surgical Journal*, January 15, 1880, contains a report by Dr. F. H. Cilley, of Barnet, Vt., of the following interesting case:

Mrs. H——, aged fifty-five, who had had dropsy during the last five years, with valvular disease of the heart, on June 18 had severe dyspnœa: she had passed no urine for twenty-four hours, and had general anasarca. Half-drachm doses of the fluid extract of jaborandi were given every four hours. Its effect was manifested in half an hour; within eighteen hours she passed sixteen pints of urine; also profuse perspiration and salivation were induced. The dyspnœa was at once relieved. A second attack was relieved by the same treatment. The patient has had tonics during the last four months, and is now in good condition.

Quebracho in Dyspnœa.—According to Dr. J. B. Berhart, of London, this new drug continues to prove itself a very efficient palliative in all forms of dyspnœa. His experience of its efficacy refers only to cases in which the dyspnœa was associated with emphysema of the lungs, atheroma of the arteries, and degeneration of the cardiac muscles. In all these cases a teaspoonful (5.00) of the liquid extract afforded *immediate relief*. In three minutes after the administration of the drug the pulse became somewhat fuller, but not increased in frequency; the patients felt their breathing easier; the face was flushed, and a gentle perspiration appeared on the forehead. There were slight drowsiness and inclination to sleep. These symptoms, however, soon subsided, while the breathing continued to be much improved.—*Medical and Surgical Reporter.*

The Treatment of Neurasthenia or Chronic Nervous Exhaustion.—In this lecture it will not be possible to enter upon a description of the treatment adapted to special forms of neurasthenia, such as the dyspeptic, the sexual, or other varieties which might be mentioned, and are actually met with in experience; I wish at present to call your attention to the medical treatment of the uncomplicated disorder.

One of the first things needful to be accomplished by medicine is to diminish reflex excitability of the nervous system, especially of the vaso-motor division of the same. For the comfort of the patient, as well as a means for recovery, it is necessary to put out of sight, as far as can be safely done, undue reflex excitability, especially of the cardiac and vaso-motor nervous apparatuses. Until this is done it will be impossible for the patient to rest as quietly as needful.

As to the medicinal agents which may be employed in these cases, I have found nothing that answers the purpose better than the milder bromides, associated with digitalis, in some such combination as the following:

| | |
|---|---|
| ℞.—Sodii Bromidi . . . . . . | ʒ vij. |
| Acid Bromohyd. (Squibb's Strong) . | ʒ iij. |
| Tr. Digitalis . . . . . . | ʒ iij. |
| Bismuth Subnit. . . . . . . | ʒ iv. |
| Syr. Prun. Virg. . . . . . . | ℥ vij.—*M.* |

*S.*—Shake well before using; one or two teaspoonfuls, before or after meals, in water.

In this prescription, as you will observe, the patient gets a small dose of the bromide of sodium, which is rendered more acceptable to the stomach, if not more useful, by the addition of bromohydric acid, especially in dyspeptic neurasthenias. The value of the prescription is enhanced by the addition of bismuth in some cases. If the acid is of the stronger kind, it should be given in proportionately smaller doses than the weaker. In such a mixture as I have offered I have reason to think partial decomposition of the subnitrate of bismuth occurs, so as to afford, probably, an acid bromide of sodium and of bismuth combined.—Dr. Jewell, in *Chicago Medical Gazette.*

ANÆMIC MENORRHAGIA.—*Editor Medical Brief:*—If Dr. S. W. Hopkins, of Bower's Mills, Mo., will try the following prescription in his anæmic menorrhagic case, I think that he will be pleased with the result. It has never failed with me:

| | |
|---|---|
| ℞.—Quin. Sulph. . . . . . . | grs. xxx. |
| Acid Phos. Dil. . . . . . . | ʒ vi. |
| Elix. Iodo. Brom. Cal. Co. . . . . | ℥ iv. |
| Tr. Gentiæ Co. . . . . . . | ℥ ij. |
| Tr. Cinchon, q. s. . . . . . . | ℥ viii. |

*M.*—*Sig.*—Teaspoonful in a little water four times a day.

Barton Dozier, M. D.

Ukiah, Cal., January 13, 1880. —*The Medical Brief.*

Attenuations.—From an old address of Prof. Armor, we clip the following, his address being textually "Medical Science and Common Sense:"

"But the high dilutions stagger our credulity, not to say our common sense, when used as curative agents. It is difficult to believe, for instance, that a patient is cured by high dilutions of lime, when he is swallowing a thousand times as much in every drink of water he takes. Every egg he eats has a thousand times as much sulphur or phosphorus in it as a 'high dilution' homeopathist would give him. Every morsel of meat he takes contains more iron than a homeopathic dose. The very air we breathe is full of these 'attenuations' in most confusing perplexity. There may be science in these high potencies, but common sense is slow to accept them."—*Obstetric Gazette.*

Collecting Bills.—Further steps toward making this a more practicable and easy process are being taken among the profession in the West (New York *Med. Journal*). The physicians of Quincy, Ill., have adopted a series of resolutions by which they agree to render their bills monthly, and employ a common collector, who will keep a delinquent-list for the benefit of his patrons.—*Louisville Med. News.*

Pruritus Ani.—During last summer I had a case of this kind which baffled all my endeavors, until I used the following prescription:—

| | |
|---|---|
| ℞.—Camphoræ. | |
| Chloral Hyd., *āā* . . . . . . | ʒ ss. |
| Ung. Petrolei . . . . . . | ʒ vij.—*M.* |

*Sig.*—Ointment.

This gave immediate relief, and a few applications only were needed; the itching was permanently allayed.

Repeated experiences with it since that time have so satisfied me of its efficiency, that I venture to suggest it to the readers of your valuable journal, in the hope that they also may find it a "friend in need." JOHN H. PACKARD, M. D.

1924 Spruce Street, Philadelphia, February 16, 1880.

—*Medical and Surgical Reporter.*

SWEATING HANDS OR FEET.—Professor Pitzer, of the American *Medical Journal*, says: "Bathe the hands and feet in alcohol at night and you will have no more trouble from sweating extremities."

### FEBRUARY REPORT OF DEATHS AND INTERMENTS IN BALTIMORE.

| CAUSES OF DEATH. | NO. | CAUSES OF DEATH. | NO. |
|---|---|---|---|
| Angina Pectoris | 3 | Fever, Typhus | 2 |
| Apoplexy | 14 | " Typho-Malarial | 3 |
| Ascites | 2 | Fracture Femur | 1 |
| Asphyxia | 5 | Gangrene, Leg | 1 |
| Asthma | 3 | Gastro Enteritis | 3 |
| Asthenia | 7 | Hernia, Strangulated | 1 |
| Atrophy | 2 | Hemorrhage of Brain | 1 |
| Atelectasis Pulmonum | 1 | " Post-Partum | 1 |
| Adynamia | 1 | Hydrocephalus | 1 |
| Bright's Disease | 9 | Inanition | 11 |
| Burns and Scalds | 3 | Inflammation of Bowels | 2 |
| Capillary Bronchitis | 4 | " Stomach | 1 |
| Collapse of Lungs | 1 | " Bronchi | 11 |
| Cancer, Breast | 3 | " Brain | 3 |
| " Liver | 1 | " Kidneys | 1 |
| " Neck | 1 | " Liver | 1 |
| " Uterus | 3 | " Pericardium | 1 |
| Casualties | 3 | " Peritoneum | 7 |
| Child Birth | 2 | " Pleura | 1 |
| Cirrhosis of Liver | 1 | " Uterus | 1 |
| Congestion of Brain | 5 | Indigestion | 1 |
| " Bowels | 1 | Intemperance | 1 |
| " Kidneys | 1 | Marasmus | 14 |
| " Lungs | 6 | Meningitis | 4 |
| " Stomach | 1 | " Tubercular | 11 |
| Consumption of Lungs | 91 | " Cerebro Spinal | 2 |
| " Bowels | 1 | Laryngeal Consumption | 1 |
| Convulsions | 25 | Mal-Nutrition | 4 |
| Compression of Brain | 1 | Malignant Pustule | 1 |
| Croup | 18 | Necrosis | 1 |
| Cyanosis | 5 | Nervous Prostration | 1 |
| Dentition | 1 | Old Age | 13 |
| Diarrhœa | 1 | Ovariotomy | 1 |
| Diphtheria | 15 | Paralysis | 12 |
| Disease of Heart | 18 | Pneumonia | 47 |
| " Liver | 1 | " Typhoid | 7 |
| " Brain | 3 | " Pleuro | 2 |
| Dropsy, General | 10 | " Broncho | 1 |
| Dysentery | 1 | Premature Birth | 4 |
| Drowned in Night Soil Liquid | 3 | Puerperal Peritonitis | 1 |
| Erysipelas | 1 | Protracted Labor | 1 |
| Fever, Intermittent | 1 | Pleural Effusion | 1 |
| " Puerperal | 4 | Scrofula | 2 |
| " Gastric | 1 | Small Pox | 1 |
| " Scarlet | 31 | Softening of Brain | 4 |
| " Typhoid | 7 | Stricture of Intestines | 1 |

### MORTUARY REPORT—(CONTINUED).

| CAUSES OF DEATH. | NO. | CAUSES OF DEATH. | NO. |
|---|---|---|---|
| Senile Catarrh | 1 | Unknown Infantile | 7 |
| Spina Bifida | 1 | Uræmia | 1 |
| Softening of Brain | 4 | Whooping Cough | 6 |
| Syphilis | 3 | | —— |
| Trismus Nascentium | 5 | Totals | 547 |
| Traumatic Entero Peritonitis | 1 | | |
| Thrush | 1 | | |
| Tumors | 4 | Annual Death Rate per 1000 during the Month | —— |
| Ulcer, Bowels | 1 | | |
| Unknown Adult | 1 | | |

### THE DEATHS REGISTERED IN THE MONTH INCLUDE

| | | | |
|---|---|---|---|
| Under 1 year | 135 | Between 30 and 40 years | 44 |
| Between 1 and 2 years | 43 | " 40 and 50 " | 37 |
| " 2 and 5 " | 45 | " 50 and 60 " | 38 |
| | —— | " 60 and 70 " | 46 |
| Under 5 years | 223 | " 70 and 80 " | 31 |
| Between 5 and 10 years | 31 | " 80 and 90 " | 11 |
| " 10 and 15 " | 12 | " 90 and 100 " | 3 |
| " 15 and 20 " | 18 | " 100 and 110 " | 2 |
| " 20 and 30 " | 51 | Above 110 years | |

### NATIVITY, ETC.

| | |
|---|---|
| Males, White | 208 |
| Females, White | 211 |
| Males, Colored | 63 |
| Females, Colored | 65 |
| | —— |
| Total | 547 |
| Still Birth | 43 |

JAMES A. STEUART, M. D.,
Commissioner of Health and Registrar.

By Order: A. R. CARTER,
Secretary.

U. S. Signal Office,
Baltimore, Md., March 1, 1880.

### METEOROLOGICAL SUMMARY FOR THE MONTH OF FEBRUARY, 1880.

| | | |
|---|---|---|
| Mean Barometer . . . . . . . . | 30.148 inches | |
| Highest Barometer . . . . . . . . | 30.688 " | on the 20th |
| Lowest " . . . . . . . . | 29.320 " | on the 3d |
| Mean Thermometer . . . . . . . | 41 degrees | |
| Highest " . . . . . . . . | 67 " | on the 29th |
| Lowest " . . . . . . . . | 15 " | on the 2d |
| Mean Relative Humidity . . . . . . | 65.0 per cent | |
| Prevailing Direction of Wind . . . . | northwest | |
| Total Movement of Wind . . . . . . | 4585 miles | |
| Highest Velocity of Wind . . . . . . | 26 miles per hour on 1st | |
| Total Rainfall, or Melted Snow . . . . | 1.96 inches | |
| Total Number of Days on which Rain or Snow Fell | 13 | |

Note.—The mean temperature of February was nearly five degrees above the average, and is the highest mean on record, although falling over one degree short of the January mean. The mean temperature of the past three winter months is 42° 2, which is also the highest mean on record, (Signal Service Record), and is 7° 4 above the average of the nine prevailing winters. The rainfall of February, 1880, falls about ¼ inch below average.

Robert Seyboth,
Observer.

Iodide of Starch in Poisoning.—As a general antidote in poisoning, Dr. Bellini, in a paper read before the Medical Society of Florence, Italy, recommends iodide of starch. It is free from any disagreeable taste, and does not possess the irritating properties of iodine, so that it can be administered in large doses. He has made numerous experiments, and states, as a result of these, that at the temperature of the stomach, and in the presence of the gastric juice, the iodide combines with many of the poisons, forming in some cases insoluble compounds, in others soluble compounds, which are harmless so long as they do not exist in too large quantities. He recommends it as safe in all cases where the nature of the poison is unknown, and as especially efficient in cases of poisoning by the alkaloids and alkaline sulphides, by ammonia, and especially by those alkaloids with which iodine forms insoluble compounds. In cases of poisoning by salts of lead and mercury, it aids the elimination of these compounds. In cases of acute poisoning, an emetic should be employed soon after the administration.—*Scientific American.*

### OF INDIVIDUAL IMPORTANCE TO EVERY ONE INTERESTED IN DENTISTRY.

I am preparing for publication "The Dental Directory of the World," and am exceedingly anxious that the names and addresses of all dentists should appear correctly in its pages.

This work will contain a general history of dentistry and its individual history in each country, written by authors of acknowledged ability. It will also contain biographical sketches of those who have been the greatest benefactors to the profession; the addresses of all the dentists in the world; the location and history of every dental college, with its corps of instructors and their duties; the dental societies, when organized, number of members and times of meeting; also the laws of each State and Country governing the practice of dentistry. It will be of great value to the dental profession as a work of reference.

I most respectfully ask all the dentists in the world; the deans of all dental colleges; the secretaries of all dental societies, and other organizations pertaining to dentistry; dealers and manufacturers of dental goods, to send to me at their earliest convenience, the necessary information (which they may have) to complete a work like the one here proposed. Any other information which they may think of value to the Directory not herein referred to, will be thankfully received.

I earnestly request every dentist in the world to send to me his or her printed or plainly written professional card containing full address, and to briefly answer the following questions:

1. What is your native tongue, and with what languages are you conversant?

2. What colleges or institutions have you attended, and what degrees have you taken?

3. Of what societies, if any, are you a member?

4. What office, if any, have you held in either?

It will be evident to all that to complete this work will require considerable time and money, both of which have already been largely expended.

I have secured copyright for the book, and it will be sold by subscription. All subscribers who send in their money before November 1st, 1880, will have their names printed in **heavy letters**, and to such addresses will most likely be sent circulars, papers, books, samples, etc., setting forth all that is new and useful to the dental profession. In order to secure your name in the Directory, you will please send the desired information *without delay*, and state whether you would like to become a subscriber.

The price of the book will be five dollars. You are not required to purchase it in order to have your name inserted.

All dental journals in the world will please copy this article for the good of the profession at large, and send me a copy. We will then give your journal ample notice in the Directory.

Hoping that this will receive favorable and prompt attention, I am,

Very respectfully,

B. M. Wilkerson, M. D., D. D. S.,

Junior Editor and Proprietor of "The Independent Practitioner,"

68 N. Charles Street, Baltimore, Md.,

United States of America.

---

### ALLEN DIE SICH FÜR DIE ZAHNARZENEIKUNST INTERESSIREN.

Im Begriff ein "Handbuch für Zahnärzte" zu publiciren, ist es von grösster Wichtigkeit, dass Name und Wohnort der darin angeführten Zahnärzte, richtig gegeben werden.

Das Werk wird eine allgemeine und special Geschichte der Zahnarzeneikunst enthalten, geschrieben von anerkannt tüchtigen schriftstellern. Ferner erscheinen biographische Skizzen der grössten Wohlthäter auf diesem Felde, so wie Wohnort aller Zahnärzte der Welt, örtliche Lage und Geschichte aller Fachschulen, mit Anführung der Herrn Lehrer und deren Pflichten; Gesellschaften zur Beförderung der Wissenschaft, wann gebildet, Zahl der Mitglieder und Zeit ihrer Zusammenkünfte, so wie die Gesetze aller Staaten und Länder, die Odontiatriepraxis betreffend. Das "Handbuch" wird in jeder Hinsicht von besonderem Werthe für die Herrn vom Fache sein.

Ich ersuche die Herrn Zahnärzte überall, die Directoren der Zahnarzeneikunstschulen, Secretäre von Gesellschaften u. anderen Organisationen zur Beförderung dieses Berufs, so wie Händler und Fabrikanten einschlägiger Instrumente, etc., mir so bald wie möglich, die für das beabsichtigte Werk nöthige Auskunft, zu übermachen, überhaupt wer den alle für das "Handbuch" nützlichen Berichte mich zu grossem Danke verpflichten.

Ich bitte jeden Zahnarzt in der Welt mir seine oder ihre gedruckte oder geschriebene, vollständige Adresse zu schicken und mir folgende Fragen in aller Kürze zu beantworten:

1. Welches ist Ihre Landessprache und mit welchen anderen Sprachen sind Sie vertraut?
2. Welche Schulen oder Institute haben Sie besucht und wo haben Sie promovirt?
3. Gehören Sie Gesellschaften an und welchen?
4. Haben Sie ein Amt in denselben bekleidet und welches?

Es ist gewiss Jedem einleuchtend, dass die Vollendung dieses Werkes, Geld und Zeit in Anspruch nehmen wird, was es denn auch schon in beträchtlichem masse gethan hat.

Das Buch, dessen Verlagsrecht ich gesichert, wird p. Subscription verkauft. Die Namen aller Subscribenten, welche den Preis vor dem 1sten November 1880 einschicken, werden in fetter Schrift gedruckt und werden an solche Adressen jedenfalls einschlägige Schriften, Bücher, Proben, etc. gesandt. Um sich seinen Namen im "Handbuch" zu sichern, wolle man die gewünschte Auskunft sofort senden und zugleich erklären ob man Subscribent zu werden wünscht.

Der Preis des Buches wird 5 Dollars betragen, jedoch bedingt dessen Ankauf, die Aufnahme eines Namens in dasselbe, keineswegs.

Zur Beförderung der Zahnheilkunde, ersuche ich alle Fachschriften höflichst, diese Ankündigung zu copiren und mir ein Exemplar davon zu schicken. Es wird solchen Journalen eine besondere Notiz im "Handbuch" gegeben werden.

In der Hoffnung, dass Obigem eine günstige und schnelle Antwort zu Theil werde, verbleibe ich,

Achtungsvoll, Ergebenst

B. M. Wilkerson, M. D., D. D. S.,

Jun. Redacteur und Eigenthümer des "Independent Practitioner,"
68 N. Charles Street, Baltimore, Md.,
United States of America.

### FAITS IMPORTANTS POUR TOUS CEUX QUI S'ENTÉRESSENT AU BON ÉTAT DES DENTS.

Je prépare pour la presse "Le Directoire dentaire du Monde," et je désire beaucoup que les noms et les adresses de tous les dentistes paraissent correctement dans ses pages.

Cet ouvrage contiendra l'histoire générale de l'art du dentiste et l'histoire dentaire particulière de chaque pays, par des ecrivains d'une capacité reconnue. Il donnera aussi des esquisses biographiques des dentistes les plus renommés; les adresses de tous les dentistes du monde; la localité et l'histoire de chaque collége dentaire, ses professeurs et leurs fonctions; les sociétés dentaires l'époque de leur organisation, le nombre de leurs membres et le temps de leurs séances; aussi que les lois qui régissent la pratique de l'art dentaire dans chaque état et dans chaque pays. Ce livre aura une grande valeur comme ouvrage de renseignements pour la profession dentaire.

Je prie respectueusement tous les dentistes du monde; les doyens de tous les colléges dentaires; les secrétaires de toutes les sociétés dentaires et autres organisations ayant rapport à l'art dentaire; les marchands et fabricants de marchandises dentaires, de m'envoyer aussitôt qu'ils le pourront commodement les renseignements nécessaires pour compléter un ouvrage comme celui que je propose. Tous les renseignements qu'ils croiraient avoir quelque utilité pour le Directoire seront reçus avec reconnaissance.

Je sollicite instamment tous les dentistes du monde de m'envoyer leur carte de profession imprimée ou distinctement écrite et de répondre briévement aux questions suivantes:

1. Quelle est votre langue maternelle et quelles langues vous sont familières?
2. Dans quels colléges ou institutions avez-vous etudié et quels diplômes avez-vous reçus?
3. Etes-vous membre d'une ou plusieurs sociétés?
4. Avez-vous eu un emploi quelconque dans ces sociétés?

Il est évident que pour compléter cet ouvrage il faut encore beaucoup de travail et d'argent malgré les dépenses dejà faites et le temps qui lui a été consacré.

Je me reserve tous les droits sur ce livre qui sera vendu par abonnement. Chaque abonné qui enverra le prix de son abonnement avant le premier Novembre, 1880, aura son nom imprimé en gros caractère ; des annonces, des journaux, des livres, des échantillons, etc., contenant tout ce qui est nouveau et utile à la profession dentaire lui seront très probablement envoyés.

Afin d'assurer à votre nom une place dans le Directoire, envoyez tout de suite s'il vous plaît les renseignements desirés et dites si vous voudriez vous abonner.

Le prix du livre sera de cinq dollars. On n'est pas obligé de l'acheter pour y avoir son nom inscrit.

Tous les journaux dentaires du monde sont priés de publier cette lettre pour le bien de la profession dentaire en général et de m'envoyer un exemplaire du numero où elle se trouvera. En retour nous publierons dans le Directoire un article assez long sur votre journal.

Espérant que la presente communication recevra de votre part une prompte et favorable attention, je suis.

Très respectueusement,

B. M. Wilkerson, M. D., D. D. S.,
Rédacteur associé et Propriétaire de "The Independent Practitioner,"
68 N. Charles Street, Baltimore, Md.,
United States of America.

---

Domestication and Brain Growth.—At the recent meeting of the British Association, Dr. Crichton Browne gave an address on the influence of domestication on brain growth. He had found by experiments that domestication had greatly reduced the brains of the duck, and he argued that men, like ducks, might be fed and housed, fenced about, and exempted from participation in the life struggle, until, like the ducks, they would depreciate in mental capacity. Their bodies might increase in size and succulence, but their brains would become straitened and withered. Disease and luxury crippled the brains. It was as true as ever that men were perfected through suffering, toil and conflict, and it was not through affluence and comfort that genuine civilization was attained. It was the civilization, not merely the domestication of mankind that must be aimed at.—*The Dental Advertiser.*

